# Characterization of the complete chloroplast genome of basswood (*Tilia mongolica* maxim.)

**DOI:** 10.1080/23802359.2021.1926358

**Published:** 2021-05-13

**Authors:** Guangshun Zheng, Yongxiu Xia, Shaofeng Li, Wenjuan Mo, Jia Guo, Jianbo Li

**Affiliations:** a National Permanent Scientific Research Base for Warm Temperate Zone Forestry of Jiulong Mountain in Beijing Experimental Center of Forestry in North China, Chinese Academy of Forestry, Beijing, China; b State Key Laboratory of Tree Genetics and Breeding, Chinese Academy of Forestry, Beijing, China

**Keywords:** Tiliaceae, *Tilia mongolica*, chloroplast genome, systematic evolution

## Abstract

Herein, we report the complete chloroplast genome of *Tilia mongolica* Maxim. from Tiliaceae. The chloroplast genome of *T. mongolica* is 162,804 bp, with a large single copy region of 91,255 bp, small single copy region of 20,355 bp, and two inverted-repeat regions of 25,597 bp. The chloroplast genome contains 130 genes, including 85 protein-coding, 8 rRNA, and 37 tRNA. The total GC content is 36.46%. The phylogenetic analysis of *T. mongolica* showed a relatively close relationship with *Tilia taishanensis*.

The Tilia family includes approximately 50 genera and 450 species distributed worldwide. They are found throughout the temperate regions of America, Asia, and Europe (Fineschi et al. 2003). *Tilia mongolica* is a species of genus the *Tilia*. It is a deciduous tree, with light gray outer bark having obvious shallow grooves and cracks, and an inner bark rich in fiber and mucus. The leaves are mostly wide ovoid, with highly serrated edges. The flowers are yellow or white, and often occur in clusters. The fruit is round or oval nut-like, having one to three seeds. They are used as wood, honey resources, and ornamental trees (Cai et al. 2015; Boo and Park 2016). Traditionally, the lime tree has belonged to its own family, Tiliaceae (Aiello 1981). However, some molecular evidence has indicated that it is closely related to other plants (Bayer et al. 1999). Therefore, owing to limited taxonomic features and frequent hybridization, the classification of lime trees has been controversial (Pigott 2012). Previous studies on the genetic classification of *Tilia* populations had focused on the use of random amplified polymorphic DNA (Colagar et al. 2013; Filiz et al. 2015) and microsatellite (Logan et al. 2015) markers. With the development of sequencing technology, chloroplast genomes have been widely used for the identification and phylogenetic analyses of plant species, including some basswoods (Cai et al. 2015; Lu et al. 2020), owing to the highly conserved genome size and sequence (Sarzi et al. 2019; Sun et al. 2019). Therefore, it was imperative for the chloroplast genome sequence of *T. mongolica* to be resolved.

The fresh leaves of *T. mongolica* were collected from Beijing, China. The samples were stored in the Experimental Center of Forestry in North China (39.970°E, 116.096°N), Chinese Academy of Forestry. A specimen was deposited at the herbarium of Experimental Center of Forestry in North China (https://www.ncbi.nlm.nih.gov/nuccore/MW386998, contact person is Guangshun Zheng and email is guangshunzheng@163.com) under the voucher number mengduan-001. A CTAB protocol was used to isolate total genomic DNA (Wang et al. 2015). Sequencing libraries were generated using a TruSeq DNA Sample Preparation Kit (Illumina, USA) and a Template Prep Kit (Pacific Biosciences, USA). Genome sequencing was performed using the Pacific Biosciences and Illumina NovaSeq platforms. A total of 21,036,342 reads (including18,275,612 high-quality reads) were obtained, and the clean reads were assembled using SPAdes (Bankevich et al. 2012) and A5-miseq (Coil et al. 2015) to construct scaffolds and contigs. The chloroplast splicing results were obtained using these software packages, and the reference genome was analyzed using Mummerv3.1 software (Kurtz et al. 2004) to determine the positional relationships among and to fill the gaps between contigs. Results were corrected using Pilonv1.18 (Walker et al. 2014) software to obtain the final chloroplast genome. The assembled complete chloroplast genome sequence’s functional annotation was performed using the online program GeSeq (https://chlorobox.mpimp-golm.mpg.de/geseq.html). Related species were used as references, and the remaining parameters were set at default values.

The chloroplast genome was annotated and submitted to GenBank (accession number MW386998). The chloroplast genome of *T. mongolica* is a typical circular DNA of 162,804 bp. It encodes two inverted repeat (IR) regions, IRa and IRb, with a large single copy (LSC) region, and a small single copy (SSC) region separating the IRs. The lengths of the LSC, SSC and IR regions are 91,255 bp, 20,355 bp, and 25,597 bp, respectively. The GC content of the chloroplast genome of *T. mongolica* is lower than the AT content, and the GC contents in the chloroplast genome, LSC, SSC, and IR regions are 36.46%, 34.06%, 31.05% and 42.90%, respectively. The chloroplast genome encodes 130 potentially functional genes, including 85 protein-coding, 8 rRNA, and 37 tRNA. rRNAs only exist in the IR region. In total, 16 genes are repeated in the IR region, 5 protein-coding, 7 tRNA, and 4 rRNA. Additionally, the *ycf1* gene is at the junction between the IR and SSC, and *rps19* is at the junction between the LSC and IR. In total, 16 genes (*trnK*, *rps16*, *trnG*, *atpF*, *rpoC1*, *trnL-UAA*, *trnV-UAC*, *petB*, *petD*, *rpl16*, *rpl2*, *ndhB*, *rps12*, *trnL-GAU*, *trnA-UGC/UCG*, and *ndhA*) contain one intron, and 2 genes (*ycf3* and *clpP1*) contain two introns. The *matK* geneis located in *trnK-UUU*, and *rps12* is a trans-spliced gene: the 5′-end is located in the LSC region, whereas the 3′-end is located in the two IR regions. The phylogenetic relationships of *T. mongolica* were estimated using the Maximum-likelihood method in MEGA7 (Kumar et al. 2016). The phylogenetic tree showed that *T. mongolica* is relatively closely ralated to the *Tilia taishanensis* (Figure 1).

**Figure 1.**
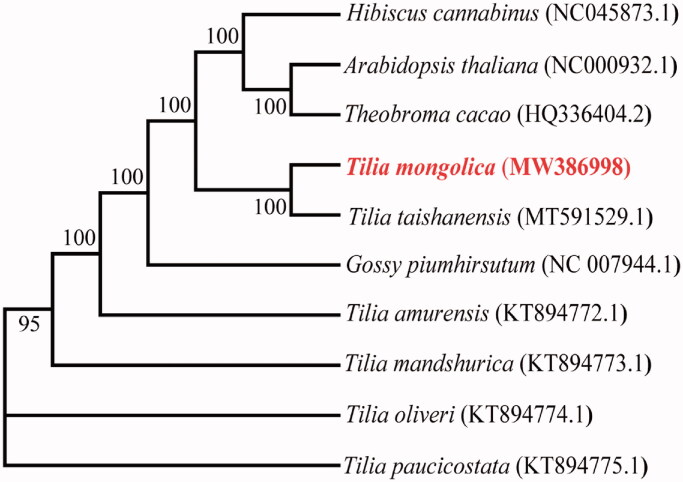
Phylogenetic tree constructed from 10 complete chloroplast genomes. The analysis was performed with the Maximum-likelihood using MEGA 7.0 software. The sequences were downloaded from NCBI GenBank, and the accession numbers are shown in parentheses.

## Data Availability

The genome sequence data that support the findings of this study are openly available in GenBank of NCBI at [https://www.ncbi.nlm.nih.gov] (https://www.ncbi.nlm.nih.gov/) under the accession number MW386998. The associated BioProject, SRA, and Bio-Sample numbers are PRJNA701724, SRR13705393, and SAMN17905928, respectively.
